# Sleep Quality and Its Determinants Among Patients With Chronic Diseases in Ethiopia: A Systematic Review With Meta‐Analysis

**DOI:** 10.1155/bmri/6736381

**Published:** 2025-12-21

**Authors:** Bekahegn Girma, Alemayehu Molla, Asresu Feleke, Takla Tamir, Ahmedin Sefa, Jemberu Nigussie

**Affiliations:** ^1^ Department of Nursing, College of Medicine and Health Science, Dilla University, Dilla, Ethiopia, du.edu.et; ^2^ Department of Psychiatry, College of Medicine and Health Science, Dilla University, Dilla, Ethiopia, du.edu.et

**Keywords:** associated factors, chronic illnesses, Ethiopia, meta-analysis, sleep quality

## Abstract

**Background:**

Patients with chronic medical and mental illnesses are more vulnerable to poor sleep quality. However, there is little aggregated evidence about poor sleep quality among this population and its determinants in Ethiopia. This study was aimed at assessing the pooled prevalence of sleep quality and its determinants among patients with chronic diseases in Ethiopia.

**Methods:**

We used the Preferred Reporting Items for Systematic Reviews and Meta‐Analyses (PRISMA) guidelines to write this review. Primary articles were retrieved from PubMed, PsycINFO, Hinari, ScienceDirect, African Journal Online (AJOL), and Google Scholar databases. A random‐effects model was applied for analysis. *I* ^2^, Cochran′s, and tau^2^ were checked to determine the degree of heterogeneity between the included studies. Egger′s test and sensitivity analysis were conducted to check publication bias.

**Results:**

The pooled prevalence of poor sleep quality among patients with chronic medical and mental illnesses was 53.12% (95% CI: 47.66, 58.58). Eight factors were associated with poor sleep quality: advanced age (POR = 1.04, 95% CI: 1.02, 1.07), female sex (POR = 2.95, 95% CI: 2.21, 3.93), social support (POR = 2.62, 95% CI: 1.90, 3.61), substance use (POR = 1.76, 95% CI: 1.51, 2.04), anxiety symptoms (POR = 2.92, 95% CI: 2.40, 3.56), comorbidity (POR: 2.47, 95% CI: 1.83, 3.33), sleep hygiene practice (POR: 2.86, 95% CI: 2.02, 4.04), and depression symptoms (POR = 3.73, 95% CI: 2.96, 4.69).

**Conclusion and Recommendation:**

More than half of patients with chronic diseases experienced poor sleep quality. Poor sleep quality was connected with advanced age, female sex, substance use, having comorbidity, inadequate social support and sleep hygiene practices, anxiety, and depression symptoms. Substance use should be restricted, and patients with chronic mental and medical illnesses should be counseled to avoid substance use. Moreover, special focus should be given to female patients, patients with other comorbid conditions, elderly individuals, and those who have poor sleep hygiene and social support. Lastly, patients with chronic medical and mental illnesses should be screened for anxiety and depression symptoms.

## 1. Background

Compared to the general population, patients with chronic illnesses are more vulnerable to poor sleep quality [1–5], especially patients with chronic illnesses such as diabetes, heart failure, chronic pain, and kidney disease who are more likely to have poor sleep quality [6]. According to primary studies, globally, around 45% of the population is affected by poor sleep quality [7–9], with a particularly high prevalence in sub‐Saharan African countries. In high‐income countries, the magnitude of poor sleep quality among patients with chronic illnesses ranges from 41% to 63.7% [10, 11]. However, in middle‐ and low‐income countries, the burden is even higher. In Ethiopia, the prevalence of poor sleep quality varied from 35.5% to 81.6% [12, 13].

Sleep is a universal requirement and a critical physiological function for survival [14, 15]. It is a state of insentience that can be disrupted by sensory or other stimuli [16]. Our body organs generate hormones to accomplish various physiological activities. However, adequate sleep quality is required to perform these functions effectively [17, 18]. A sleep duration of 7–9 h is recommended for young people and adults and 7–8 h for elderly individuals [19].

A person′s level of energy, activity, and readiness for the day is reflected in their sleep quality, which encompasses both qualitative and quantitative elements. Quantitative components include sleep length, sleep latency, and the sense of restfulness upon waking, while qualitative factors include the depth and peacefulness experienced upon arousal [20]. The Pittsburgh Sleep Quality Index (PSQI) is a standardized test used to measure the quality of sleep; a score of less than five indicates poor sleep [21]. Many facets of health are affected by low sleep quality [22].

Poor sleep quality increases the risk of cognitive decline, cardiovascular diseases, Type 2 diabetes, and mental health issues [1–5]. It also contributes to accidents, prolonged hospital stays, reduced productivity, and economic costs, with global estimates attributing billions of dollars annually to sleep‐related losses. Ultimately, poor sleep is a significant risk factor for increased morbidity and all‐cause mortality, underscoring its public health importance [4, 6–10]. More specifically, in patients with chronic illnesses, poor sleep quality exacerbates physical symptoms, impairs immunological function, and increases inflammation. It also leads to poor treatment adherence and mental health problems. These consequences lower quality of life and hasten the progression of disease [23].

Sleep disturbances can be caused by long‐term conditions such as diabetes, asthma, and heart failure. Diabetes produces nocturnal hypoglycemia and discomfort, which disrupt sleep [1], while heart failure causes sleep disturbances due to symptoms like shortness of breath [2]. Coughing at night is one of the symptoms of asthma that reduces the quality of sleep [3]. Consequently, inadequate sleep makes many disorders worse by increasing inflammation and compromising immunological function [4, 5]. Therefore, identifying coexisting sleep problems among patients with chronic illnesses will lead to better treatment outcomes [24].

Previous studies identified some determinants, such as occupational status, educational status [13], gender [25], low income, longer disease duration, poor disease control, and marital status as direct determinants of poor sleep quality among chronic patients [11, 26–29].

The prevalence of poor sleep quality was assessed in different countries and at different times. In Ethiopia, there are systematic reviews conducted among diabetic patients only [30, 31] and on patients with chronic illnesses [32]. However, their findings were unsummarized, disease‐specific, and did not identify determinants of poor sleep quality for patients with chronic medical and mental illnesses. Therefore, this gap underscores the need for a comprehensive, up‐to‐date, and methodologically sound review to summarize existing evidence and identify key determinants of sleep quality in this vulnerable population.

Enhancing the quality of sleep is essential for lowering mortality because it lowers the chance of developing chronic illnesses [33]. Therefore, this review and meta‐analysis attempt to aid in the creation of policies that promote the Sustainable Development Goal (SDG), which calls for a 25% reduction in preventable deaths from noncommunicable diseases by 2025, as established by the United Nations [34]. Furthermore, this review will help policymakers in developing strategies to improve sleep quality among patients with chronic illnesses and reduce the risk of associated health complications and may indirectly benefit patients with chronic medical and mental illnesses. Lastly, it will help healthcare providers by providing the pooled prevalence of poor sleep quality and its factors.

## 2. Research Questions

• What is the pooled prevalence of poor sleep quality among Ethiopian patients with chronic diseases?
• What are the determinants of poor sleep quality among patients with chronic diseases in Ethiopia?

## 3. Method and Material

This review and meta‐analysis was registered in PROSPERO with the ID CRD42023213501.

### 3.1. Eligibility Criteria

#### 3.1.1. Included Studies

##### 3.1.1.1. Population

Articles conducted among patients with chronic diseases such as DM, HTN, HIV, CHF, schizophrenia, cancer, and epilepsy aged 18 years and older were included.

##### 3.1.1.2. Outcome

Articles reported the prevalence of the outcome variable, poor sleep quality, that was included in them.

##### 3.1.1.3. Study Settings

Primary studies conducted across different regions of Ethiopia up to June 01, 2026, were encompassed.

##### 3.1.1.4. Study Design

Observational studies with original data that reported at least the prevalence of poor sleep quality among patients with chronic medical and mental illnesses or associated factors were included. This encompassed cohort, case‐control, and cross‐sectional studies.

##### 3.1.1.5. Publication Status

Both published and unpublished articles were considered in this review.

#### 3.1.2. Excluded Studies

Studies like trial, systematic reviews, mini reviews, reports, and case studies were excluded from this review. Moreover, articles that did not report the outcome variable as a proportion or did not report the associated factors using odds ratios were not included.

### 3.2. Information Source and Search Strategy

This systematic review and meta‐analysis was carried out in accordance with the PRISMA 2020 guidelines [35]. These studies, both published and unpublished (gray literature), that address the extent of poor sleep quality in Ethiopia and its associated factors were reviewed in this study. AJOL, PsycINFO, Hinari, ScienceDirect, PubMed/MEDLINE, and Google Scholar were retrieved for relevant articles.

Additionally, we scanned the reference lists of included studies to identify other relevant works not found in the databases. For Objective 1, we used search terms such as “magnitude” OR “prevalence” OR “burden” AND “sleep quality” AND “chronic illnesses” OR “diabetic patients” OR “HIV” OR “heart failure” OR “hypertension” OR “epilepsy” AND “Ethiopia”. For Objective 2, we used “determinants” OR “predictors” OR “factors” AND “sleep quality” AND “chronic illnesses” AND “Ethiopia”. We selected the above chronic diseases because they are among the most prevalent conditions in Ethiopia.

We limited our search to English‐language studies published between February 1, 2010, and June 1, 2026, in order to yield relevant evidence. We used EndNote version X8 software to manage citations and remove duplicates.

#### 3.2.1. Study Risk of Bias Assessment

To evaluate the risk of bias in the included studies, the Newcastle–Ottawa Scale (NOS), adapted for the cross‐sectional studies, was employed [36]. The studies were assessed based on the NOS criteria: selection (five stars), comparability (two stars), and outcome (three stars); a maximum score of 10 (stars) [36]. B.G. and J.N. independently assessed the studies using the adapted NOS. The scale evaluated 10 quality criteria, and studies scoring at least six out of 10 were included in the review and meta‐analysis. The reason for this cutoff point was that studies which had below 6 were considered unsatisfactory.

### 3.3. Effect Measures

This review and meta‐analysis had two objectives. The first objective was to determine the pooled prevalence of poor sleep quality among patients with chronic medical and mental illnesses in Ethiopia. This was calculated by dividing the number of patients with poor sleep quality by the total number of patients and multiplying by 100. The second objective was to assess the variables associated with poor sleep quality in Ethiopian patients with chronic medical and mental illnesses. In this study, factors reported as determinants of poor sleep quality in at least three studies were considered for the meta‐analysis. Pooled odds ratios (PORs) were used to compute the pooled effect.

### 3.4. Synthesis Methods

Data extracted from each study was imported into Stata Version 16 for analysis. A random‐effects model was employed in the meta‐analysis because the included studies varied significantly from one another. The standard error for the proportion in each original study was determined using the binomial distribution formula. Subgroup analysis was conducted to identify sources of heterogeneity [37–39]. The effects of selected associated factors on the quality of sleep were also investigated in this review and meta‐analysis. We used text, tables, forest plots, and POR with 95% CI to present the results. Heterogeneity between studies was evaluated using the *I* ^2^ statistic, and values of less than 50%, between 50% and 75%, and greater than 75% were classified as low, moderate, and high heterogeneity, respectively [40]. Moreover, Cochran′s *Q* test and tau^2^ were conducted. Sensitivity analysis was conducted to identify influential or outlier studies.

### 3.5. Reporting Bias Assessment

Begg′s and Egger′s statistical tests [41] and funnel plot [42] were done to indicate evidence of publication bias. A *p* value of < 0.05 was used to declare the occurrence of evidence for publication bias.

## 4. Results

### 4.1. Study Search and Selection

**Figure 1:**
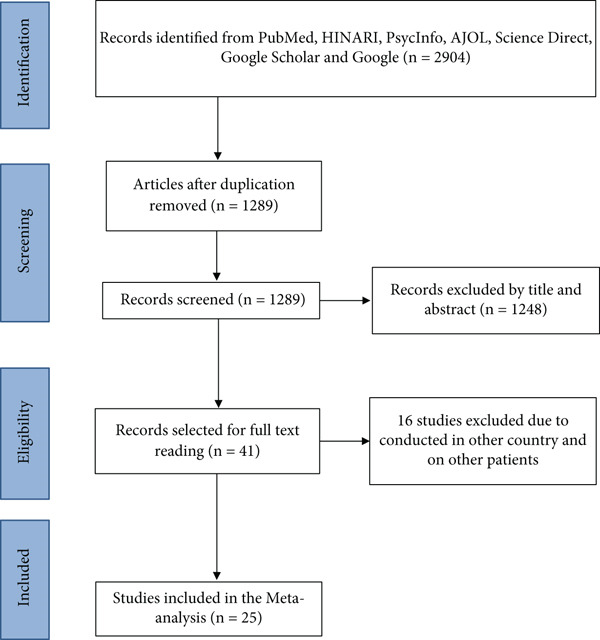
Flow diagram of the studies included in the review of sleep quality in Ethiopia, 2025.

Our search was limited to full‐text human studies conducted between February 2010 and June 1, 2026. A total of 2904 primary articles were retrieved from PubMed, PsycINFO, Hinari, Science Direct, AJOL databases, and as well as Google Scholar and Google. Of the total, 1615 articles were excluded due to duplication, and 1248 were excluded based on their titles and abstracts. Only 41 studies were selected for a full reading. However, an additional 16 studies were excluded due to being conducted out of Ethiopia because they were conducted outside Ethiopia, were systematic reviews, or were conducted in the general population. Lastly, a total of 25 articles that fulfilled the inclusion criteria were selected for meta‐analysis, as shown in Figure 1.

### 4.2. Characteristics of the Included Articles

Our review and meta‐analysis was conducted among 9326 patients with chronic illnesses. Except for one, all the included studies were cross‐sectional and conducted in four regions: Amhara [27, 43–54], Oromia [13, 26, 29, 55–58], SNNP [56], and Addis Ababa [11, 25, 59, 60]. Among the included studies, the smallest sample size was from a study done in Jimma, Ethiopia (99 participants) [57], while the largest sample size was from a study conducted in the Amhara Region, Ethiopia (565 participants).

Twenty‐three (92%) of the included studies were conducted within 5 years (2020–2025). Thirteen of these were conducted in the Amhara Region, Ethiopia, while only one study was conducted in SNNP, Ethiopia.

Fifteen (60%) of the included studies used a systematic sampling technique to select study participants. Furthermore, the highest (81.6%) and lowest (35.5%) prevalence of poor sleep quality were reported in studies conducted in the Oromia Region, Ethiopia. Lastly, all included studies used the PSQI tool to assess sleep quality with a similar cut point (Table 1).

**Table 1:** Characteristics of the included articles for poor sleep quality among chronic medical and mental patients in Ethiopia, 2025 (*n* = 25).

**Authors**	**Region**	**Publication year**	**Design**	**Sampling**	**Sample size**	**Response rate (%)**	**Prevalence**	**Population**	**Risk of bias**	**Tool**
Getahun et al.	Oromia	2021	IBCSS	Simple	111	100	37.8	HF	8	PSQI
Jemere et al.	Oromia	2019	IBCSS	Consecutive	99	100	55.6	DM	9	PSQI
Birhanu et al.	Amhara	2020	IBCSS	Systematic	430	100	47.2	DM	7	PSQI
Adem et al.	AA	2020	IBCSS	Systematic	423	98.1	65.5	Epilepsy	9	PSQI
Edmealem et al.	Amhara	2020	IBCSS	Stratified	396	97.7	36.5	Chronic illnesses	8	PSQI
Bedaso et al.	SNNP	2020	IBCSS	Systematic	422	92.1	57.6	HIV	9	PSQI
Mengistu et al.	AA	2021	IBCSS	Systematic	408	97.1	55.6	HIV	7	PSQI
Metekiya et al.	Oromia	2020	IBCSS	Systematic	410	100	59	HIV	9	PSQI
Abdu and Dule	Oromia	2020	IBCSS	Systematic	341	98.53	57.1	HIV	8	PSQI
Birhanu et al.	Oromia	2020	IBCSS	Systematic	279	100	35.5	HTN	9	PSQI
Abera et al.	Oromia	2015	IBCSS	Conventional	297	93.6	81.6	HF	8	PSQI
Edmealem A et al.	Amhara	2021	IBCSS	Stratified	344	100	36	Chronic illnesses	8	PSQI
Dule et al.	Oromia	2020	IBCSS	Consecutive	422	97.4	57.4	Schizophrenia	7	PSQI
Abebe et al.	AA	2023	IBCSS	Systematic	264	93.61	53.79	Cancer	7	PSQI
Adane et al.	Amhara	2022	IBCSS	Systematic	399	95.80	55.1	HIV	7	PSQI
Ayanaw et al.	Amhara	2022	IBCSS	Systematic	563	96.9	37.7	HTN	8	PSQI
Endeshaw et al.	Amhara	2022	IBCSS	Systematic	410	97	71.5	Cancer	8	PSQI
GebreEyesus et al.	Amhara	2023	IBCSS	Systematic	419	100	36	HIV	7	PSQI
Legas et al.	Amhara	2022	IBCSS	Simple	411	97.1	39.4	HIV	7	PSQI
Simie Tsega et al.	Amhara	2021	IBCSS	Systematic	565	98.30	68.8	Epilepsy	7	PSQI
Tadesse et al.	AA	2023	IBCSS	Systematic	388	93.94	73.7	HIV	7	PSQI
Zewdu et al.	Amhara	2022	IBCSS	Cluster	292	100	50.7	DM	7	PSQI
Gela et al.	Amhara	2024	IBCSS	Systematic	424	100	42.9	CKD	8	PSQI
Gete et al.	Amhara	2025	IBCSS	Consecutive	406	100	64.5	Asthma	9	PSQI
Ayehu et al.	Amhara	2025	Cohort	Whole sampling	403	100	50.1	Stroke	8	PSQI

Abbreviations: AA, Addis Ababa; CKD, chronic kidney disease; DM, diabetic mellitus; HF, heart failure; HIV, human immunodeficiency virus; IBCS, institution‐based cross‐sectional study; PSQI, Pittsburgh Sleep Quality Index; simple, simple random sampling; SNNP, South Nation Nationalities and People; stratified, stratified random sampling; systematic, systematic random sampling.

### 4.3. Results of Syntheses and Reporting Bias

**Figure 2:**
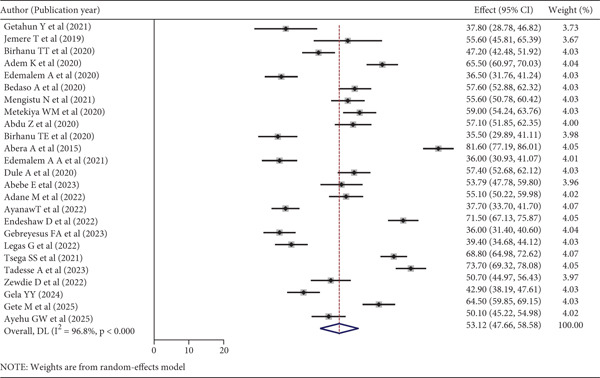
Forest plot of the included studies to determine the pooled magnitude of poor sleep quality among chronic medical and mental patients in Ethiopia, 2025 (*n* = 25).

To estimate the pooled prevalence of poor sleep quality among patients with chronic medical and mental illnesses, 25 studies were included. In this review and meta‐analysis, the pooled prevalence of poor sleep quality among patients was 53.12% (95% CI: 47.66, 58.58). The heterogeneity of the included studies was high (*I* ^2^ = 96.80*%*, *p* < 0.01, tau^2^ = 186.7246, and Cochran′s *Q* = 749.18). Due to this heterogeneity, we used a random‐effects model to estimate the pooled magnitude of poor sleep quality (Figure 2).

To explore the source of heterogeneity, subgroup analyses were conducted by region, population type, and sample size. High heterogeneity was observed among studies conducted in the Oromia and Amhara regions (*I* ^2^ = 96.9*%*, *p* < 0.01). Based on sample size, studies with fewer than 370 participants showed high heterogeneity (*I* ^2^ = 97.30*%*, *p* < 0.01), as did studies focusing on heart failure patients (*I* ^2^ = 98.6*%*, *p* < 0.01). A high prevalence of poor sleep quality was reported in studies conducted among epileptic patients (67.38%) and in Addis Ababa (62.27%) (Table 2) (Supporting Information 1: File S1).

**Table 2:** Subgroup analyses for poor sleep quality among chronic medical and mental patients in Ethiopia, 2025 (*n* = 25).

**Determinants**	**Categories**	**Number of studies**	**Prevalence (95% CI)**	**Heterogeneity**	
				**I** ^2^ **(%)**	**p** **value**
Region	Oromia	7	55.03 (43.02, 66.03)	96.9	< 0.01
	Amhara	13	48.97 (41.66, 56.28)	96.9	< 0.01
	Addis Ababa	4	62.27 (53.16, 71.38)	92.80	< 0.01

Sample size	> 370	17	54.04 (47.90, 60.19)	96.8	< 0.01
	< 370	8	51.08 (38.51, 63.65)	97.30	< 0.01

Population	Chronic illnesses	2	36.27 (32.80, 39.73)	0	0.88
	HIV	8	54.19 (45.63, 62.75)	96.10	< 0.01
	DM	3	49.75 (45.73, 53.78)	22.30	0.276
	HF	2	59.88 (16.96, 102.81)	98.6	< 0.01
	HTN	2	36.96 (33.70, 40.22)	0	0.532
	Epilepsy	2	67.38 (64.18, 70.59)	16.1	0.275

Abbreviations: DM, diabetic mellitus; HF, heart failure; HIV, human immunodeficiency virus; HTN, hypertension.

**Figure 3:**
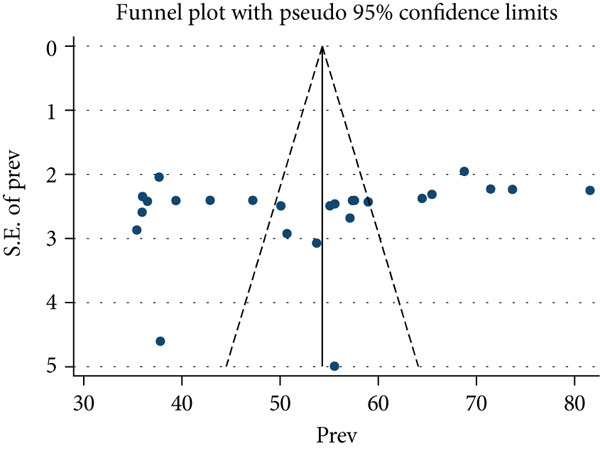
Funnel plot showing distribution of primary studies included in this study, 2025 (*n* = 25).

**Figure 4:**
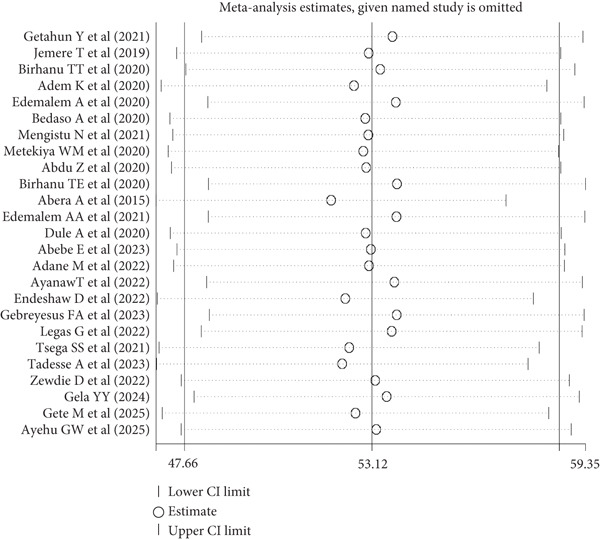
Sensitivity analysis for studies included to assess the pooled prevalence of poor sleep quality among chronic medical and mental patients in Ethiopia, 2025 (*n* = 25).

To assess publication bias, both a funnel plot and Egger′s test were used. Based on the funnel plot, most of the studies were outside the expected range (Figure 3). However, Egger′s test was not significant (*p* = 0.227), indicating no evidence of publication bias. Finally, the sensitivity analysis showed no outlier studies that significantly affected the pooled prevalence estimate (Figure 4).

### 4.4. Determinants of Poor Sleep Quality Among Chronic Patients

Variables identified as factors for poor sleep quality in at least three primary studies were considered for this meta‐analysis. Based on this, eight variables—advanced age, female sex, poor social support, anxiety symptoms, poor sleep hygiene practices, depressive symptoms, comorbidity, and substance use—were identified as associated factors for poor sleep quality.

The pooled odds of poor sleep quality among female patients were 2.95 times higher than those of males (95% CI: 2.21, 3.93). Patients with depressive and anxiety symptoms had 3.73 (95% CI: 2.96, 4.69) and 2.92 (95% CI: 2.40, 3.56) times higher odds of poor sleep quality, respectively, compared to their counterparts.

In addition, patients aged over 65 years had a 1.04 times higher risk of poor sleep quality compared to those younger than 65 years (95% CI: 1.02, 1.07). Patients who used substances (alcohol, khat, or cigarettes) were 1.76 times more likely to experience poor sleep quality compared to those who did not use substances (95% CI: 1.51, 2.04).

Furthermore, patients with comorbid diseases had a 2.47 times higher risk for poor sleep quality compared to those without comorbid conditions (95% CI: 1.83, 3.33). Patients with poor social support were also more likely to develop poor sleep quality (POR: 2.62; 95% CI: 1.90, 3.61). Lastly, patients with poor sleep hygiene practices had higher odds of poor sleep quality (POR: 2.86; 95% CI: 2.02, 4.04) than those with good sleep hygiene practices (Table 3) (Supporting Information 2: File S2).

**Table 3:** Meta‐analysis for determinants of poor sleep quality among chronic medical and mental patients in Ethiopia, 2025 (*n* = 25).

**Determinants**	**Number of studies**	**OR (95% CI)**	**Heterogeneity**	
			**I** ^2^ **(%)**	**p** **value**
Age	6	1.04 (1.02, 1.07)	89.30	< 0.01
Sex	4	2.95 (2.21, 3.93)	0	0.881
Substance use	6	1.76 (1.51, 2.04)	59.3	< 0.031
Anxiety symptoms	9	2.92 (2.40, 3.56)	42.60	< 0.01
Depression symptoms	8	3.73 (2.96, 4.69)	67.9	< 0.003
Comorbidity	6	2.47 (1.83, 3.33)	0.756	< 0.01
Sleep hygiene practice	3	2.86 (2.02, 4.04)	0	0.58
Social support	5	2.62 (1.90, 3.61)	0	0.883

## 5. Discussion

In this systematic review and meta‐analysis, the pooled prevalence of poor sleep quality among patients with chronic diseases was 53.12% (95% CI: 47.66, 58.58). Advanced age, female sex, presence of comorbidities, anxiety symptoms, poor social support, depressive symptoms, poor sleep hygiene practices, and substance use were significant factors associated with poor sleep quality among patients with chronic medical and mental illnesses. However, almost all of the included studies were cross‐sectional (overestimation of the findings should be avoided).

The pooled prevalence was higher compared to a systematic review and meta‐analysis conducted in low‐ and middle‐income countries (33.2%) [61] and a study done in China (41.5%) [62]. This might be because our study included patients with chronic medical and mental illnesses, who are more vulnerable to poor sleep quality [1, 2]. However, it was similar to a review done in Ethiopia (53%) among the general population [63] and a global meta‐analysis conducted among cancer patients (57.4%) [64]. This similarity might be due to comparable populations and methodological factors, such as the use of similar tools to assess sleep quality across studies.

The pooled prevalence of poor sleep quality reported in our analysis was low compared to studies conducted in Iran (77% [65] and 73.8% [66]), Canada (77.2%) [67], and a global meta‐analysis [64]. This disparity might be due to differences in healthcare systems, and the two Iranian studies were conducted among patients with chronic kidney disease (on dialysis), who are at higher risk for poor sleep quality compared to the patients included in our study. Additionally, the variation might be attributed to differences in the study periods, as there has been greater focus on mental health in recent years.

Patients with chronic illnesses and advanced age had more risk for poor sleep quality. This finding was supported by studies conducted in Poland [68] and China [69]. This similarity could be due to the combined impacts of chronic illness and aging, which interfere with the circadian and neuroendocrine regulation of sleep. Polypharmacy, functional deterioration, and frequent discomfort all impede restful sleep. Furthermore, sleep disruptions are made worse by the high rates of anxiety and sadness in this population.

In this study, female patients with chronic illnesses had a high risk for poor sleep quality. This finding was supported by studies done in Spain [70] and Nigeria [71]. This could be because females are more susceptible to stress due to the affliction of excessive household responsibility, which can contribute to sleep disturbances [72]. Furthermore, sleep quality is known to be impacted by hormonal changes associated with the menstrual cycle, pregnancy, and menopause [73]. Lastly, women are more susceptible to poor sleep quality due to genetic factors that also contribute to sleep problems, such as serotonin‐related genes (5‐HTTLPR) and circadian clock genes [74].

Patients with chronic medical and mental illnesses and who had anxiety symptoms had a higher risk of poor sleep quality. This result was similar to previous studies conducted in Iran [75], Brazil [76], the United States [77], and Korea [10]. This similarity could be due to a bidirectional relationship between poor sleep quality and anxiety symptoms. In a vicious cycle, anxiety can lead to increased arousal, making it more difficult to fall asleep, and a lack of sleep might further worsen anxiety, given that sleep problems can stem from both physical and mental health conditions [78].

The current study also found that patients with chronic medical and mental illnesses who had depression symptoms had a higher risk of having poor sleep quality compared to nondepressed patients. This finding was similar to studies conducted in the United States [79], Norway [80], India [81], and China [82]. This may be due to a decrease in neurotransmitters such as serotonin in depressed patients, which results in diminished cognitive performance that directly affects sleep patterns. It may also be because depression affects the entire sleep cycle, further impairing sleep quality.

In the present study, chronic patients who were substance abusers had a higher risk of poor sleep quality. This finding was supported by a study conducted in Finland [83] and by a general knowledge that substance use directly impacts various central nervous system processes and there is a bidirectional relationship between sleep quality and substance use [84].

Patients with another comorbid disease had a higher risk for poor sleep quality as compared to their counterparts. This result was in line with studies conducted in Australia [85], Turkey [86], and China [20]. This similarity may be due to neurological dysregulation and increased systemic inflammation. Chronic illnesses frequently cause long‐lasting inflammatory reactions that disrupt sleep–wake cycles, and mental health disorders worsen neurochemical imbalances that are essential for controlling sleep. Furthermore, psychological anxiety and intricate treatment plans further interfere with the continuity of sleep. For this susceptible group, the combination of biological and psychological variables poses a significant obstacle to getting a good night′s sleep [87, 88].

Furthermore, poor social support was significantly associated with poor sleep quality. This finding was supported by studies conducted among Chinese cancer survivors [89, 90]. This resemblance may result from the lack of practical and emotional support, which can exacerbate worry, tension, and loneliness, all of which have a detrimental effect on sleep. Social support improves emotional stability and coping skills, which act as a buffer. Patients are more susceptible to sleep disruptions and psychological anguish in the absence of this care [88].

Lastly, poor sleep hygiene practices were significantly associated with poor sleep quality. This finding was supported by a study conducted in Nigeria [91, 92]. The similarity may result from unstable sleep patterns, excessive screen time, and the exacerbation of prebedtime stimulating activities. These actions disrupt circadian cycles and decrease the effectiveness of sleep. Therefore, in a group that is already at risk, poor sleep hygiene makes sleep issues much worse.

Even though our study was a review and meta‐analysis, it has some limitations. Firstly, because the included articles were cross‐sectional studies, it was difficult to establish a temporal relationship between the outcome and independent variables. For example, the bidirectional association between poor sleep quality and mental health symptoms could not be examined, whether poor sleep exacerbates mental health issues or vice versa. Secondly, there was high heterogeneity among the included studies, which may affect the precision of the pooled estimates. Lastly, our review was limited to studies conducted in Ethiopia, which may restrict the generalizability of the findings to other countries or contexts.

## 6. Conclusion

In Ethiopia, more than half of patients with chronic medical and mental illnesses had poor sleep quality. Advanced age, female sex, substance use, anxiety and depression symptoms, having comorbidity, and poor social support and sleep hygiene practices were significant determinants of poor sleep quality. These findings highlight the need for a comprehensive, integrated approach to chronic care that addresses both physical and mental health components.

The Federal Ministry of Health, pertinent professional associations, and designers of chronic care programs ought to give priority to incorporating mental health screening and evidence‐based psychological therapies into the management of chronic illnesses in light of these findings. In particular, it is advised to use cognitive behavioral therapy for insomnia (CBT‐I), CBT for distress associated with chronic illness, and CBT for anxiety and depression symptoms. These treatments promote long‐term health outcomes, lessen psychological discomfort, and enhance the quality of sleep. In order to address the modifiable behavioral and social factors that contribute to poor sleep, counseling therapies should also incorporate teaching about sleep hygiene and the improvement of psychosocial support.

Additionally, physicians and other healthcare professionals should regularly evaluate the quality of sleep, screen for signs of depression and anxiety, and integrate sleep screening into chronic disease clinics. They should also include appropriate behavioral interventions in their care plans and pay particular attention to vulnerable categories, such as women, the elderly, and people with comorbid diseases. Moreover, the screening of sleep quality should be integrated into the chronic disease clinics, and digital interventions should be considered. Furthermore, to further improve the general well‐being and quality of life of people with chronic illnesses, focused health education and community initiatives should work to prevent and minimize substance use. Lastly, future researchers should prioritize longitudinal designs and context‐specific interventions.

**Nomenclature**
CI: confidence interval
OR: odds ratio
SNNP: South Nations and Nationalities People
USA: United States of America
WHO: World Health Organization

## Ethics Statement

The authors have nothing to report.

## Consent

The authors have nothing to report.

## Disclosure

All authors read and approved the final version of the manuscript to be considered for publication.

## Conflicts of Interest

The authors declare no conflicts of interest.

## Author Contributions

B.G. and J.N. conceived the idea and participated in data extraction, analysis, and draft writing. A.M., A.F., T.T., and A.S. participated in analysis and manuscript preparation and revision.

## Funding

No funding was received for this manuscript.

## Supporting Information

Additional supporting information can be found online in the Supporting Information section.

## Supporting information


**Supporting Information 1** File S1: Subgroup analysis results showing variations in pooled estimates across different study characteristics and populations.

**Supporting Information 2** File S2: Analysis output of factors associated with poor sleep quality among patients with chronic diseases in Ethiopia.

## Data Availability

The data included in this study is available and can be accessed by contacting the corresponding author.
