# Supplementary material for: Sleep Quality and Its Determinants Among Patients With Chronic Diseases in Ethiopia: A Systematic Review With Meta‐Analysis
Source: Biomed Res Int. 2025 Dec 21;2025:6736381. doi: 10.1155/bmri/6736381 (PMC12719613; doi:10.1155/bmri/6736381)
Supplement: Supplementary file 1 — Supporting Information 1 File S1: Subgroup analysis results showing variations in pooled estimates across different study characteristics and populations. [file BMRI-2025-6736381-s001.docx]

**Supplementary material 1 sub group analysis**

**Supplementary material 1:** Subgroup analysis based on region for sleep quality of patients with chronic diseases in Ethiopia, 2025

**Supplementary material 1:** Subgroup analysis based on sample size for sleep quality of patients with chronic diseases in Ethiopia, 2025

**Supplementary material 1:** Subgroup analysis based on disease type for sleep quality of patients with chronic diseases in Ethiopia, 2025
