# Supplementary material for: Sleep Quality and Its Determinants Among Patients With Chronic Diseases in Ethiopia: A Systematic Review With Meta‐Analysis
Source: Biomed Res Int. 2025 Dec 21;2025:6736381. doi: 10.1155/bmri/6736381 (PMC12719613; doi:10.1155/bmri/6736381)
Supplement: Supplementary file 2 — Supporting Information 2 File S2: Analysis output of factors associated with poor sleep quality among patients with chronic diseases in Ethiopia. [file BMRI-2025-6736381-s002.docx]

**Supplementary material 2**: Factors associated with poor sleep quality among patients with chronic diseases in Ethiopia, 2025

1. Age

1. Sex

1. Depression

1. Anxiety

1. Substance use

1. Co-morbidity

1. Sleep hygiene practice

1. Social support
